# Dr. Marisa Holubar on the “Mentorship Mindset” …promoting learning, building partnerships, and finding fulfillment

**DOI:** 10.1017/ash.2023.412

**Published:** 2023-11-06

**Authors:** Marisa Holubar

**Affiliations:** 1 Department of Quality, Patient Safety and Effectiveness, Stanford Health Care, Stanford, CA, USA; 2 Division of Infectious Diseases and Geographic Medicine, Stanford University School of Medicine, Stanford, CA, USA

**1. Tell us about the Stanford Antimicrobial Safety and Sustainability Program (SASS). How does this program advance the agenda of stewardship in the US and abroad? What was your path to becoming a SASS medical director and what are some of the partnerships that have made you successful?**

SASS is our healthcare system’s antimicrobial stewardship program; we adopted this name to communicate our overarching priorities more precisely within our institution—namely optimizing patient safety as well as promoting antimicrobial sustainability. Our group’s fundamental values include collaboration and creativity—we view antimicrobial stewardship with a wide lens. I believe these fundamental values are key to our program’s success and critical for professional fulfillment and combating burnout. This approach has also allowed us to successfully interact with partners in the U.S. as well as internationally with the latter focusing on low- and middle-income countries. All success is shared among the entire team, most notably our stellar and indefatigable infectious diseases pharmacists—David Ha, Emily Mui, Lina Meng, and Will Alegria. Working with the four of them is simply the best part of my job.

I was drawn to antimicrobial stewardship because it centers my interests: solid clinical care, medical education, data, and behavior change. My career in stewardship grew in part from my global health experience, including the year I spent working as a clinician in Western Kenya at AMPATH and Moi Teaching and Referral Hospital prior to starting my fellowship. I gravitate toward work that systematically impacts health care beyond my individual patient interactions as a physician. At their best, stewardship and global health programs support both patients and healthcare professionals within a system and thus impact public health. I’m also intrigued by the complex medical culture that drives antibiotic prescribing—and enjoy the diligent work of influencing behavior change, building relationships, and connecting with diverse audiences—all of which are core to effective antimicrobial stewardship.

**2. Tell us more about your collaborations with the World Health Organization and the California Department of Health. How did you launch these collaborations, and what advice do you have for readers seeking to expand the scope of their work in similar ways?**

My colleagues, Drs. Stan Deresinski and Elizabeth Robilotti, developed a free online course describing the foundations of antimicrobial stewardship, from concept to program development, in 2012. This course caught the attention of our colleagues in the WHO-Europe regional office. From there, I led the development of another free, online case-based course for the WHO that targets frontline clinicians and demonstrates how stewardship principles can be incorporated into daily practice. This course was designed to reinforce a solid clinical foundation for the management of common infections and supplement local stewardship efforts; it has subsequently been translated into multiple languages. In 2016, we became the first WHO Collaborating Center in Antimicrobial Stewardship, and in this role, we support policy and protocol development as well as educational outreach efforts. Over the past 8 years, we have partnered with the WHO to promote stewardship in many countries, including Armenia, Uzbekistan, Jordan, and Ukraine. Learning about healthcare systems across the world and supporting their stewardship efforts has informed and elevated our program at Stanford in countless ways.

My engagement with the California Department of Health was more straightforward. I applied for membership on CDPH’s Healthcare-Associated Infections Advisory Committee at the suggestion of my mentors and ultimately served as chair. During my tenure, we explored antibiotic use in dentistry, developed a toolkit to support ASP implementation in skilled nursing facilities, and launched an ASP honor roll that also connects programs across the state. I learned a tremendous amount from this experience, especially about the shared challenges of healthcare institutions across the state and how local and state public health departments can promote optimal practice.

Each of these activities are quite different. But fundamentally, the stewardship community is full of interesting, engaging, and passionate people whose professional interests likely align closely with yours. My advice is to ask your mentors and colleagues about rewarding opportunities and experiences they would recommend you pursue and network!

**3. You’ve seamlessly merged your passion and expertise in medical education and stewardship and helped launch specialized training programs for ID fellows pursuing these careers. Tell us about this work and why it’s so critical to the future of our profession. What basic Med-Ed skills should all stewards, IPs, and epidemiologists possess to do our jobs well? How can we better integrate Med-Ed principles into our daily work?**

As I’m sure ASHE readership will agree, antimicrobial stewardship physician leaders require a unique skill set that extends beyond clinical expertise in infectious diseases. I direct our ID fellowship program’s ASP track which we launched in 2020 to give interested fellows protected time to develop additional leadership and administrative skills. Our ASP fellows become integral and valued members of our multi-disciplinary team and amass experience ranging from active participation in patient-directed activities like ICU handshake rounds to planning for and leading new long-term projects. Each ASP fellow’s experience is unique based upon their interests and what projects we’ve prioritized—which makes directing this program even more rewarding for me. I love working with a fellow to craft their time with us to accumulate varied experience, hone specific skill sets, and establish ASP “street cred” so that they are competitive on the job market and are well-positioned to launch their career.

Education—and influencing learners and colleagues—is implicit in many of our daily activities. Exceptional educators spend time crafting their message, honing their content and delivery to ensure that key points are clear, pithy, and memorable. They plan how best to connect with their learners. And afterward, they humbly and systematically evaluate a session and reflect on which of their key points resonated and which did not. Strategically connecting with our audiences, especially when providing unsolicited advice and education, and building in time for reflection can help all of us to continue to hone our med-ed skills.

These principles are also critical when developing effective curriculum and setting powerful educational agendas. I just joined the SHEA antimicrobial stewardship track chairs, and the guiding principles of our planning sessions include strategically connecting with a diverse audience and maximizing each session’s impact and relevance to new antimicrobial stewardship leaders as our field continues to evolve.

**4. What are the biggest internal drivers of your success?**

I enjoy building things—programs, projects, and professional relationships—and I’m motivated by the quality of the end product. Knowing that I’m contributing to the welfare of both current and future patients is rewarding. I’m also highly motivated by the success (defined broadly) of our group as a whole and the individuals we train.

**5. Describe a pivotal mentor relationship that altered the trajectory of your career and helped get you to this stage**

I can’t pick just one. Dr. Stan Deresinski, with whom I’ve worked closely for almost a decade, has influenced my career in countless ways. He has been both a mentor and sponsor—gently nudging me when I stalled (often because of self-doubt) before walking through doors he opened for me. He is a great thinker and writer and always pushes me to set my sights higher. Drs. Lucy Tompkins and Upi Singh have also been incredible mentors and role models. Both women are trailblazers, and their energy and passion for infectious diseases is quite literally contagious. Upi is a visionary—she dreams big, sees opportunity in each challenge and unique promise in every individual. She recognized and celebrated my passion for mentorship early and continues to help me craft my career so that this is a central focus. She also taught me how to negotiate for myself. Lucy is a natural and gifted sponsor. She never misses an opportunity to promote me and tout my accomplishments to others. Her support helped me establish local credibility early—especially as I transitioned from fellow to junior faculty at the institution in which I trained. I try to emulate these three people every day. I, along with many others, am immensely grateful for the confidence and belief they have in me.

**6. What are the rewards of training and mentoring ID fellows and junior faculty into stewardship and IP careers? How has this benefited you personally?**

Mentoring ID fellows is one of **the best** parts of my job. Each fellow I’ve mentored brings unique experience, interests, and energy to our relationship. I love brainstorming and crafting their fellowship with them so that they can develop their unique niche—the choices are endless. The transition from resident to fellow to first professional job is definitely hard but is also fascinating and rewarding. I learn a tremendous amount from each fellow—about our field and about myself. I’ve actively worked on my mentoring skill set over the last several years and derive a significant amount of professional and personal fulfillment and joy from this role. I’m incredibly proud of every single fellow I’ve mentored. One such leader I had the privilege to mentor is Dr. Sharon Ong’uti, our first official ASP track graduate, who joined Vanderbilt University as the ASP medical director for two of their regional hospitals and is now their ID fellowship’s associate program director.

**7. What were some barriers you’ve encountered in your career and how did you address them?**

Like many junior faculty, I overextended myself early in my career. I had a hard time saying no to any opportunity that came my way. You never know where it might lead and what doors it may open. Early in your career, you also don’t have sufficient context to evaluate what opportunities are right for you until you’ve tried some that aren’t the best fit. But taking on too much meant that I did not feel I was able to do everything to my own standards. Over time, I have intentionally become more diligent about evaluating the opportunity cost of pursuing new things. I’ve also learned to not make rash decisions. My advice to junior colleagues is to almost always graciously ask for time to consider the pros and cons of a new endeavor. If I know that an opportunity is not right for me but seems promising, I offer it to one of my junior colleagues—but only after I’ve vetted it and can see how it will help them advance their individual career goals.

**8. What career advice would you share with young professionals in stewardship and IP?**

Start slow and pace yourself. There are many ways to do these jobs well—and have a satisfying career. Get super clear about your personal values and priorities—this will make it a lot easier to rationally evaluate opportunities and turn them down if they don’t align with what is most important to you. Take advantage of leadership courses locally and outside of your institution, especially those that center self-reflection and assessment and balance. Stanford Department of Medicine offers a fantastic leadership series called Making SPACE, designed and led by the inspiring and tireless Rebecca Merrill. I use and teach skills I learned in SPACE every single day and am extremely grateful to the community of local leaders that continue to grow out of this program.

**9. What outside activities or hobbies do you rely on to help you adjust to the challenges of your work?**

I work hard to maintain boundaries between work and home to protect my time with my husband and children. We are an outdoors family—we love to hike, ski, and travel, especially to national parks. As an introvert, reading fiction is restorative and rejuvenating for me; it is my “go-to” self-care ritual. I read every day, even if it’s just a few pages.

**10. Finally, which non-medical book, essay, or podcast has impacted you professionally that you’d recommend to our readers?**

This is my very favorite of your great questions! I read for escape, certainly, but also to learn. And there is so much to learn from fiction. Reading also builds empathy, open-mindedness, and creativity. Many of my favorite books depict characters I often think about and whose traits or experiences I see in other people. Some recent, incredible examples include “The Love Songs of W.E.B. Dubois” by Honorée Fanonne Jeffers, in which one character struggles with addiction, and “I am Lucy Barton” by Elizabeth Strout, whose main character recounts her childhood growing up in poverty. “Calling for a Blanket Dance” by Oscar Hokeah and “The Consequences” by Manuel Muñoz also fit this bill. But I could go on and on and on…. Reading about other people’s experiences helps me build what I value most in my career—connection and relationships with other people. And because it so effectively helps me re-charge, reading allows me to continue to craft the career and life I want.

